# Childhood Socioeconomic Status Does Not Predict Late-Life Cognitive Decline in the 1936 Lothian Birth Cohort

**DOI:** 10.3389/fpsyg.2021.679044

**Published:** 2021-06-21

**Authors:** Stéphanie Racine Maurice, Alisone Hébert, Valérie Turcotte, Olivier Potvin, Carol Hudon, Simon Duchesne

**Affiliations:** ^1^Faculté des Sciences Sociales, Êcole de psychologie, Université Laval, Quebec, Canada; ^2^CERVO Brain Research Center, Centre Intégré Universitaire en Santé et Services Sociaux de la Capitale Nationale, Quebec, Canada; ^3^Département de radiologie et médecine nucléaire, Faculté de médecine, Université Laval, Quebec, Canada

**Keywords:** cognitive aging, cognition, early life, health status disparities, life span, socioeconomic status

## Abstract

This study examined childhood socioeconomic status (SES) as a predictor of later life cognitive decline. Data came from 519 participants in the Lothian Birth Cohort 1936 (LBC1936) study. SES measures at 11 years of age included parental educational attainment, father’s occupational status, household characteristics and a composite measure of global childhood SES (i.e., a total of low SES childhood indicators). Cognitive abilities were assessed by the Mini-Mental State Exam at ages 69.8, 72.8 and 76.7 years. Most indicators of low childhood SES (i.e., father manual worker, less than secondary school father education, household overcrowding, exterior located toilet, and global childhood SES) did not predict cognitive decline between the ages of 69.8 and 76.7. Participants with less educated mothers showed an increase in cognitive decline (*β* = −0.132, *p* = 0.048, and CI = −0.80, −0.00). The relationship between maternal educational attainment and cognitive decline became non-significant when controlling for adult SES (i.e., participant educational attainment and occupation). Adult SES did not mediate the latter relationship. This study provides new evidence that childhood SES alone is not strongly associated with cognitive decline. New knowledge is critical to improving population health by identifying life span stages in which interventions might be effective in preventing cognitive decline.

## Introduction

A major challenge for cognitive aging researchers has been to understand the causes of age-related decline in cognitive function. Thus, a better understanding of risk factors contributing to diverging aging trajectories would increase the effectiveness and efficiency of identifying at-risk individuals of cognitive decline, enabling earlier intervention aimed at maintaining cognitive capabilities into older age.

Aging trajectories can be shaped by multiple environmental and individual factors (Livingston et al., 2020), some of which can be viewed as a risk for both normal and pathological cognitive decline (Plassman et al., 2010; Baumgart et al., 2015). Cognitive reserve is among the factors that could determine the resilience to the latter. For instance, previous studies have reported that a weaker cognitive reserve (Stern, 2002; Stern et al., 2019), suggested by lower educational level or lower occupational attainment during adulthood, was associated with subsequent cognitive decline in later life (Lamballais et al., 2020). Though studies in adulthood are abundant, a paucity of research on risk factors during childhood remains, which means that their impact is not as well ascertained. Therefore, the association between childhood socioeconomic status (SES) and cognitive decline is the subject of current debate among researchers.

Socioeconomic status is a complex and multidimensional construct that can be conceptualized and measured in various ways. Indicators generally represent access to material and social resources and assets, rank within a social-economic hierarchy, or both (Matthews and Gallo, 2011; Brito and Noble, 2014). All levels of SES are linked to different degrees of stress exposure, community poverty, as well as physical, emotional, and cognitive health disparities (Hackman and Farah, 2009). Children from higher-SES households have access to additional resources promoting optimal development (e.g., cognitively stimulating home environments, healthier nutrition) than their lower-SES counterparts for whom exposure to developmental risk is increased, notably exposure to chronic stress (Duncan and Magnuson, 2003). Additionally, SES is one of the most important deciding factors when it comes to the fundamental quality of a social environment. In turn, social conditions influence exposure to chronic stress as well as other social conditions associated with disease (Link and Phelan, 1995).

As per a recent meta-analysis by Wang et al. (2019), childhood SES should not be underestimated, as childhood SES is associated with disrupted brain development and cognitive decline in later life. Some studies proffered that childhood SES influences neurocognitive development (Richards and Hatch, 2011) and is associated with global cognition (Cermakova et al., 2018) as well as cognitive decline in old age (Marden et al., 2017). A longitudinal study conducted by Melrose et al. (2015) using the UC Davis Aging Diversity Cohort (*N* = 333; *Mage* = 75.0 years) revealed that participants from lower childhood SES percentile rankings present the fastest rate of global cognitive decline over a 10-year period. However, the conclusions presented by Wang et al. (2019) are challenged by a previous critical review concluding that evidence for an association with pathological decline is yet to be clearly determined (Seifan et al., 2015).

Part of the latter inconsistency arises from the widespread difference in measurement and operationalization of SES as a risk factor among studies, contributing to the absence of a consensus (Maccora et al., 2020). When estimating childhood SES, family-related indicators such as parental educational attainment, parental occupation, income, and housing characteristics are to be considered (Matthews and Gallo, 2011; Jednoróg et al., 2012).

### Parental Educational Attainment

A study by Rogers et al. (2009) found that participants over 70 years of age with less educated mothers (<eighth grade) had a 2.0 odds ratio of cognitive impairment, even after adjusting for paternal educational attainment (*N* = 892). After including individual participant’s educational attainment into the analysis model, the odds ratio for cognitive impairment of participants with less educated mothers resulted in 1.6 (Rogers et al., 2009). In a different study, cognitive decline over a 12-year period was shown to be reduced among participants whose mothers had more than 8 years of education (*N* = 9,407; over 65 years of age; adult SES covariate variable; Lyu and Burr, 2015). In contrast, another study showed that individuals over 51 years of age with highly educated parents (i.e., superior to a secondary school diploma) presented higher levels of initial cognitive functioning but not of cognitive decline (*N* = 8,833; *Mage* = 73.9 years; adult SES covariate variable; González et al., 2013). These diverging results are possibly partially explained by coding or analysis disparities (Rogers et al., 2009; Brito and Noble, 2014).

### Income and Parental Occupation

Income offers a direct relation between a family’s access to material resources and health. The impact of income on one’s health can be explained by access to quality resources (i.e., housing, nutrition) as well as health and educational facilities. In absence of income data, parental occupation is a good proxy, as it is strongly associated with income (Galobardes et al., 2006a). Depending on the scale, parental occupations are categorized into levels or classes ranking from higher to lower status (Galobardes et al., 2006b). Lee et al. (2003) analyzed the cognitive abilities of women between 70 and 79 years of age within a 2-year interval. Results showed that women whose fathers were farmers have a slightly higher odds ratio of global cognitive decline as compared with white-collar professionals. However, opposite results were found in a study using data from the Lothian Birth Cohort 1936 study (LBC1936) including numerous sociodemographic, fitness, health, and genetic predictors of age-related cognitive decline. In the latter study, childhood SES was established by using the Scottish Index of Multiple Deprivation scores as well as participant’s and father’s occupation, showing no association with cognitive decline between 70 and 76 years of age (Ritchie et al., 2016).

### Housing Characteristics

Combining housing characteristics such as household crowding (i.e., number of household occupants per room) and household amenities (e.g., interior or exterior toilets) to income or occupational data increases SES childhood accuracy by illustrating how material resources are utilized by family members (Galobardes et al., 2006a). Though less commonly used in health studies, outdoor toilets are an indication of lower SES in early 20th century Scotland (Banham, 1997). For example, a study using the LBC1936 included toilet location as an indicator when analyzing the correlation between childhood SES (i.e., paternal and participant educational attainment, paternal occupation and a composite variable composed of household crowding and toilet location) and intelligence (Johnson et al., 2010). Traditionally, household crowding ratios ranging from >1 to >2 occupants per room are overcrowding standards (Marshy, 1999). For instance, a study showed no association between childhood SES (i.e., composite variable: housing crowding and number of books, *N* = 20,244) and cognitive aging, based on housing conditions as an indicator of childhood SES with overcrowding defined as two occupants or more per room (Cermakova et al., 2018). However, another study demonstrated that individuals from low childhood SES (i.e., household size of seven or more occupants and fathers with manual occupation; *N* = 574) had a 2.8 odds ratio of pathological cognitive decline in later life (Moceri et al., 2001). These results point out the operationalizing heterogeneity in childhood SES measures when analyzing cognitive decline.

The current study expands on prior research by examining if childhood SES (i.e., parental occupation, parental educational attainment, household crowding, and toilet location) predicted cognitive decline in later life. It was hypothesized that individuals from lower childhood SES (i.e., father manual worker, less than secondary school father education, less than secondary school mother education, household overcrowding, and exterior located toilet) would show a significant reduction of cognitive functioning in later life.

## Materials and Methods

### Data

**Figure 1:**
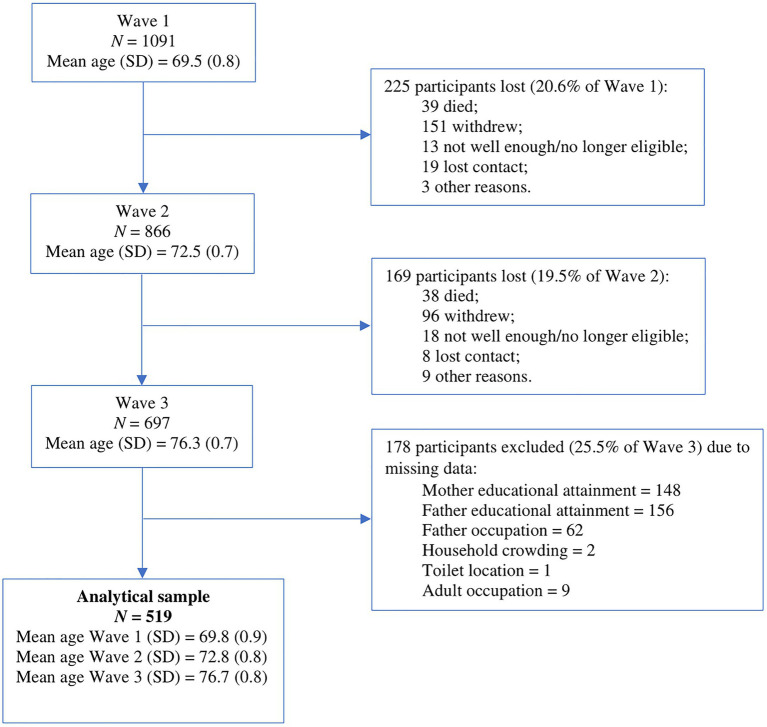
Flow chart of the analytical sample derivation across waves of testing and attrition in the Lothian Birth Cohort 1936 (LBC1936) study. Adapted from Figure 2 in Taylor et al. (2018).

Lothian Birth Cohort 1936 is a longitudinal study offering a substantial amount of cohort data (Deary et al., 2007, 2012; Taylor et al., 2018). The study was initiated in 2004 by sending mailed letter invitations to surviving members of the Scottish Mental Survey of 1947 (*N* = 70,805) who resided in the City of Edinburgh and the surrounding Lothian region of Scotland. The Scottish Mental Survey applied a valid test of general intelligence to all 11-year-old children born in 1936 and attending Scottish schools in June 1947 (Scottish Council for Research in Education, 1949). A total of 1,091 participants were recruited into Wave 1 of the LBC1936 study (543 women; *Mage* = 69.5 years). Participants gave full and informed written consent before partaking in the LBC1936 study. They were assessed on five different occasions between 2004 and 2019, with follow-up occurring every 3 years on average. Following initial assessments in Wave 1, rates of attrition have been approximately 20% between each wave, mainly as a result of participant death and withdrawal due to health conditions or chronic incapacity (Taylor et al., 2018). Also, participants who dropped out had lower Mini-Mental State Exam scores at all three waves as well as lower adult socioeconomic status (Taylor et al., 2018). For this study, participants that showed available data for every childhood SES indicator (i.e., parental occupation, parental educational attainment, household crowding, and toilet location), at least one Mini-Mental State Exam score at Wave 1 or Wave 2, and a Mini-Mental State Exam score from Wave 3 were included into the analytical sample (Figure 1). Ethical approval was obtained from Multi-Centre Research Ethics Committee for Scotland (MREC/01/0/56; Wave 1), the Lothian Research Ethics Committee (LREC/2003/2/29; Wave 1), and the Scotland A Research Ethics Committee (07/MRE00/58; Waves 2–5). The authors assert that all procedures contributing to this work comply with the ethical standards of the relevant national and institutional committees on human experimentation and with the Helsinki Declaration of 1975, as revised in 2008.

### Measures of Childhood Socioeconomic Status

#### Participants’ Sociodemographics

Data such as the participant’s birth date, gender, educational attainment, and adult main occupation were obtained in a structured initial interview (Deary et al., 2007).

#### Parental Sociodemographics

Data on household crowding at 11 years of age (i.e., number of house occupants and number of rooms), father’s occupation and individual parental educational attainment (i.e., number of years of full-time education) were collected in a questionnaire administered in the weeks prior to participant cognitive testing at Wave 1.

#### Parental Social Class

Table 1 summarizes the Census 1951 Classification of Occupations of General Register Office (1956) used by the LBC1936 study to provide an indication of childhood socioeconomic position based on paternal occupation. The Classification of Occupations aims to differentiate economic positions within the labor market and social positions within the organizational structure. Within this grouping, manual workers are mainly classified as having a lower supervisory and technical occupation (class 4) or a semi-routine and routine occupation (class 5).

**Table 1:** Parental socioeconomic status (SES) classes of the study sample, based on the Census 1951 Classification of Occupations of General Register Office (1956).

Class number	Class description
1	Higher managerial, administrative, and professional occupations
2	Intermediate occupations
3	Small employers and own account workers
4	Lower supervisory and technical occupations
5	Semi-routine and routine occupations

### Measures of Adult Socioeconomic Status

Measures of participant educational attainment (i.e., number of years of full-time education) and occupation were included as covariate variables in this study. The Classification of Occupations of Office of Population Censuses and Surveys (1980; Table 2) was used as a benchmark to measure participants’ SES during adulthood. Within the five occupational classes, manual workers are usually classified in manual occupations (class 3.5), partly skilled occupations (class 4), and unskilled occupations (class 5).

**Table 2:** Adult socioeconomic status classes of the study sample, based on the Classification of Occupations of Office of Population Censuses and Surveys (1980).

Class number	Class description
I	Professional occupations
II	Managerial and technical occupations
III	Skilled occupations (N) Non-Manual (M) Manual
IV	Partly skilled occupations
V	Unskilled occupations

### Measures of Adult Cognitive Function

The LBC1936 team assessed participants of the LBC1936 study at five different times of assessment, between ages 70 and 82. However, only data from Waves 1–3 were used in this study. The first assessment referred to as Wave 1 (2004–2007, *Mage* = 69.5 years), included measuring initial cognitive function by administering the Mini-Mental State Exam (Taylor et al., 2018). The Mini-Mental State Exam was then used again at Wave 2 (2007–2010, *Mage* = 72.5 years) and at Wave 3 (2011–2013, *Mage* = 76.3 years) to monitor cognitive aging (Taylor et al., 2018). The Mini-Mental State Exam has a test-retest reliability of 0.98 (Folstein et al., 1975) with a minimal change in scores attributable to practice effects (Tombaugh, 2005).

### Statistical Analyses

The current longitudinal study has a simple design, analyzing cognitive decline based on two-time points (i.e., the strongest Mini-Mental State Exam score obtained in Wave 1 or 2 compared to the score obtained in Wave 3) and has no missing data in the final analytical sample. The relationship between childhood SES and cognitive decline was assessed using regression analysis, performed with IBM’s SPSS Statistics Base software (Version 26; IBM Corp, 2019), and tested with an alpha level of 0.05. Descriptive statistics were calculated based on gathered sociodemographic data. The hypothesis of this study was tested by conducting multiple linear regressions, using the General Linear Model function. Most predictors were significantly correlated with one another, though a matrix correlation excluded the presence of perfect multicollinearity. In preparation for analysis, predictors were coded into dichotomous variables: (a) father educational attainment: medium to high = 0 (>9 years of school) vs. low = 1 (≤9 years of school); (b) father’s occupation: non-manual = 0 (classes 1–3) vs. manual = 1 (classes 4–5); (c) toilet location: interior = 0 vs. exterior = 1; and (d) household overcrowding: absence of overcrowding = 0 (<2 occupants per room excluding toilets) vs. overcrowding = 1 (≥2 occupants per room excluding toilets). Finally, a composite childhood SES variable was conceptualized to measure individual global childhood SES by summing predictors into a total score for each participant. Individual total scores ranged from 0 to 5 and a higher score meant a lower global childhood SES.

Covariate variables were coded in the following manner: (a) participant educational attainment: medium to high = 0 (>9 years of school) vs. low = 1 (≤9 years of school); (b) participant occupation: non-manual = 0 (classes 1–3) vs. manual = 1 (classes 3.5–5).

The dependent variable, cognitive decline, was determined by subtracting the greater of the first two Mini-Mental State Exam scores obtained (Wave 1 or 2) from Mini-Mental State Exam scores obtained in Wave 3. Higher negative variance represents greater cognitive decline. Results reflect changes in individual cognitive performance between 69.8 (*SD* = 0.90) and 76.7 (*SD* = 0.76) years of age within our analytical sample. The mean time between Waves 1 and 2 was 2.98 years (*SD* = 0.28), and 3.77 years (*SD* = 0.28) between Waves 2 and 3. From Waves 1–3, the mean time was 6.75 years (*SD* = 0.31), with a minimum of 5.12 years and a maximum of 8.98 years (Taylor et al., 2018, p. 2). The median interquartile range of the study sample is 3.5 months between Waves 1 and 2 and 1.9 months between Waves 2 and 3.

## Results

### Sociodemographics

Within the analytic sample of 697 participants, 178 were excluded due to missing data, resulting in a final sample composed of 519 individuals (256 women, 49.3%). The age of participants, on average, at each wave after the exclusion of those with missing data was 69.8 years (*SD* = 0.89), 72.8 years (*SD* = 0.79), and 76.7 years (*SD* = 0.76), respectively. Also, participants, on average, obtained 10.9 years of education (*SD* = 1.2). This is equivalent to a secondary school diploma in early 20th century Scotland (Knox, 2000). Only 2.7% of participants were classified as less educated. Most participants came from households in which both mothers and fathers had completed 9 years or less of education. Only 17.7% of participants had an occupation classified as manual (i.e., classes 3.5–5). As displayed in Table 3, only 17.5% of participants had fathers classified as manual workers (i.e., classes 4 and 5). Finally, 18.7% of participants were exposed to household overcrowding whereas 11.4% used an exterior toilet as a child. Sociodemographic data collected from excluded individuals is shown in Table 3.

**Table 3:** Descriptive statistics of indicators of low childhood SES.

Variables	Analytic sample (*n* = 519)	Excluded participants (*n* = 178)
	*n* (%)	*n* (%)
Mother low educational attainment	290 (55.9)	17 (60.7)
Father low educational attainment	306 (59.0)	10 (50.0)
Father manual worker	91 (17.5)	15 (13.2)
Household overcrowding	97 (18.7)	27 (15.5)
Exterior toilet	59 (11.4)	16 (9.1)
Global childhood SES		
1 indicator	65 (12.5)	
2 indicators	172 (33.1)	
3 indicators	87 (16.8)	
4 indicators	37 (7.1)	
5 indicators	5 (1.0)	

Participants were excluded from the analytic sample due to missing data; Values are expressed as valid percentages.

### Childhood Socioeconomic Status and Future Cognitive Decline

Results failed to show that most indicators of low childhood SES (i.e., father manual worker, less than secondary school father education, household overcrowding and exterior located toilet, global SES) predicted cognitive decline between the ages of 69.8 and 76.7 (Table 4). However, main effect results supported that participants with less educated mothers (i.e., less than secondary school mother education) showed an increase in cognitive decline between the ages of 69.8 and 76.7 years of age (*β* = −0.132, *p* = 0.048, and CI = −0.80, −0.00). As a recent study showed that low childhood SES had a greater association to cognitive decline in women (*N* = 84,059; *Mage* = 64.0 years; Wolfova et al., 2021), we analyzed gender as a possible moderator between participants with less educated mothers and cognitive decline. Results showed no interaction between gender and less educated mothers within our analytical sample (*β* = −0.001, CI = −0.529, 0.522). When computing adult SES covariate variables into the main effect statistical model, the *p*-value moved from 0.048 to 0.053. Consequently, the relationship between less educated mothers and cognitive decline became non-significant (*β* = −0.129, CI = −0.79, −0.01; Table 5). This led to further investigation by performing a mediation analysis of the relationship between low parental educational attainment and cognitive decline. To do so, Pingouin statistics software was used (Vallat, 2018). Mediation results showed that the direct relationship between less educated mothers and cognitive decline remained significant (*B* = −0.340, SE = 0.17, and CI = − 1.75, 0.40) and the indirect relationship between less educated mothers, adult SES and cognitive decline was insignificant (low adult educational attainment: *B* = −0.090, SE = 0.92, and CI = −1.76, 0.40; adult manual worker: *B* = −0.047, SE = 0.13, and CI = −0.43, 0.17). The indirect relationship between less educated fathers, adult SES and cognitive decline was also not statistically significant in our sample: (low adult educational attainment: *B* = −0.097, SE = 1.02, and CI = −1.86, 0.29; adult manual worker: *B* = −0.053, SE = 0.11, and CI = −0.41, 0.08). Thus, results did not support adult SES as being a mediator between childhood SES and cognitive decline. Additionally, we tested for the possible interaction between having a highly educated mother but living in low SES conditions in relationship to a participant’s cognitive decline within the analytical sample. Results showed no association with cognitive decline in older age.

**Table 4:** Multiple regression analysis results for cognitive decline based on childhood SES among individuals aged 69.8 and 76.7 years.

Predictor^a^	*Β*	*β*	*p*	*t*	95% CI
Mother low educational attainment^b^	−0.401	−0.132	0.048	−1.982	−0.80, −0.00
Father low educational attainment^b^	0.142	0.046	0.485	0.698	−0.26, 0.54
Father manual worker^b^	0.340	0.085	0.060	1.888	−0.01, 0.69
Overcrowding^b^	0.169	0.044	0.345	0.945	−0.18, 0.52
Exterior toilet^b^	−0.311	−0.065	0.152	−1.435	−0.74, −0.12
Global childhood SES^c^	−0.032	−0.028	0.525	−0.636	−0.13, 0.07

*N* = 519; SES, socioeconomic status; CI, confidence interval.

a Dichotomous variables.

b *R*^2^ = 0.019;

c *R*^2^ = 0.001.

**Table 5:** Multiple regression analyses results for cognitive decline^a^ based on childhood SES with adult SES covariates among individuals aged 69.8 and 76.7 years.

Predictor^b^	*Β*	*β*	*p*	*t*	95% CI
**Individual indicator model^c^**					
Mother low educational attainment	−0.394	−0.129	0.053	−1.941	−0.79, 0.01
Father low educational attainment	0.142	0.046	0.487	0.695	−0.26, 0.54
Father manual worker	0.344	0.087	0.057	1.906	−0.01, 0.70
Overcrowding	0.187	0.048	0.305	1.026	−0.17,0.55
Exterior toilet	−0.316	−0.066	0.147	−1.452	−0.74, 0.11
Adult manual worker	−0.079	−0.020	0.657	−0.444	−0.43, 027
Participant’s low educational attainment	−0.160	−0.017	0.697	−0.390	−0.97, 0.65
**Global childhood SES model^d^**					
Global childhood SES	−0.029	−0.025	0.575	−0.561	−0.13, 0.07
Participant manual worker	−0.052	−0.013	0.770	−0.319	−0.40, 0.30
Participant low educational attainment	−0.132	−0.014	0.750	−0.319	−0.94, −0.68

*N* = 519; SES, socioeconomic status; CI, confidence interval.

a Adult SES as covariate (i.e., participant educational attainment and adult occupation).

b Dichotomous variables.

c *R*^2^ = 0.020;

d *R*^2^ = 0.001.

## Discussion

We expanded on prior research by increasing the quantity and subcategories of childhood SES indicators, offering a more accurate estimation of individual childhood SES, and focusing on cognitive decline. Consistent with previous research, initial results indicated that participants with less educated mothers predicted greater decline in the eighth decade of life (Kaplan et al., 2001; Rogers et al., 2009; Lyu and Burr, 2015). Mother’s educational attainment was the only indicator of childhood SES associated with cognitive decline. However, when controlling for adult SES, the association was slightly reduced and became non-significant. These results are coherent with results from previous studies (González et al., 2013; Greenfield and Moorman, 2019) but contradict the results of others such as Lyu and Burr (2015). Furthermore, results from a mediation analysis failed to provide evidence that adult SES explained the relationship between childhood SES and cognitive decline in later life. These findings suggest that childhood SES alone does not have a strong association with cognitive decline in old age. Thus, untangling the link between childhood SES and cognitive decline requires a better understanding of the role of adult SES within a life course perspective.

To link the influence of childhood SES on cognition, three models have been generally proposed in the literature (Cable, 2014). The sensitive period model, a direct effect model, stipulates that experiences occurring within sensitive periods of cerebral development may induce permanent structural and functional alterations increasing the risk of neuropathology in old age (Bornstein, 1989; Panchanathan and Frankenhuis, 2016). The pathway model is a mediating effect model (Gilman and McCormick, 2010; Matthews and Gallo, 2011) stipulating that adult SES explains the relationship between childhood SES and health in later life (Matthews and Gallo, 2011). The hypothesis of this study applied to the first concept, the critical period model, as by controlling for adult SES, the direct effect of childhood SES on cognitive decline was examined. On the other hand, the analysis of adult SES as a mediator of the indirect relationship between childhood SES and cognitive decline reflected the pathway model. Findings did not support the possible proffered role of adult SES in the critical period model or the pathway model. However, distinct childhood and adult SES roles were not investigated using the cumulative risk model.

The accumulation model suggests that the intensity and duration of exposure to socioeconomic hardship can have a cumulative influence on cognitive health (Cohen et al., 2010). In this model, total exposure to low SES is important. It is likely that being exposed to both low childhood SES and adult SES increases the risk of cognitive impairment much more than experiencing low SES only in childhood or adulthood (Graham, 2002). A subcategory of the accumulation model is the social mobility model in which movements between SES positions across the life span, such as moving from low childhood SES to high adult SES, can modify the prediction of health outcomes in later life (Cohen et al., 2005; Cable, 2014). The initial association between mother’s educational attainment and cognitive decline is possibly explained by the fact that mothers with higher education are more likely to provide stimulating cognitive environments to their children when compared to less educated mothers (Guo and Harris, 2000). It is also possible that the influence of low SES on cognitive decline is not associated with reduced access to material resources but rather with parental characteristics influencing parent-child interactions (Erola et al., 2016). In this study, LBC1936 participant’s mothers were more likely to oversee the home environment than later generations, as 39% of mothers of the analytical sample were classified as housewives. Moreover, studies have shown that parental educational attainment is correlated to children’s educational attainment in adulthood, with a greater correlation with the mother’s educational attainment (Korupp et al., 2002; Conley, 2008; Buis, 2013). However, Scotland implemented a policy in 1936 making it mandatory for all children to attend school until the minimal requirement for a secondary school diploma was obtained (i.e., 9 years of education; Knox, 2000). This created an upward social mobility shift between parental educational attainment and participant’s educational attainment for the cohort of LBC1936, reducing the amount of less educated participants in adulthood in contrast to their parents within the analytical sample. It is possible that Scotland’s new education policy altered the cumulative risk’s influence on later cognitive health and contributed to better cognitive reserve. This is important as cumulative influences on cognitive health may be influential across an individual’s life span and across generations (Jones et al., 2019). The accumulation risk model is a venue to be explored further in subsequent studies. Furthermore, with childhood SES also expressing its effect through access to quality nutrition (Duncan and Magnuson, 2003), adding early body growth measures (e.g., leg length, height, and head circumference) as indicators would complete childhood SES estimation and increase its accuracy in regards to late-life cognitive outcomes (Seifan et al., 2015; Tom et al., 2020). Finally, LBC1936 being a longitudinal study, Mini-Mental State Exam scores have also been collected at Wave 4 (*Mag*e = 79.0 years) and Wave 5 (*Mage* = 82.0), albeit in a smaller sample. Thus, a subsequent study examining the effect of childhood SES on cognitive decline during these two periods of later-life might provide different results.

## Strengths and Limitations

Longitudinal birth cohorts, such as LBC1936, guard against cohort effects by collecting multiple measures over time from a single set of participants who grew up in similar life conditions (Sattler et al., 2012). Moreover, this study used a larger sample size, increasing statistical power and result reliability. However, the current longitudinal study also reflects some degree of sampling bias. Participants who continued within the testing process were more cognitively able as dropouts showed lower Mini-Mental State Exam scores at all three waves (Taylor et al., 2018). Furthermore, apart from low parental educational attainment, descriptive statistics of the analytical sample point out that participants from low childhood SES and low adult SES are underrepresented. Attrition data also highlighted that dropout participants displayed lower adult SES (Taylor et al., 2018). One potential explanation is sample selectivity; individuals from disadvantaged childhoods are less likely to volunteer in a study. A methodological disadvantage is that childhood SES predictors were based on retrospective reports, increasing the risk of recall bias, a possible source of error for childhood SES measures (Hardt and Rutter, 2004).

## Conclusion and Implications

This study contributes empirical evidence to the body of research on childhood SES as a predictor of late-life cognitive decline. Analysis performed did not find supporting evidence that childhood SES is associated with cognitive decline when controlling for adult SES. Additional analysis did not support the mediator role of adult SES. Building from this, future studies should aim to investigate in more depth the complex life course psychosocial pathways linking childhood SES, adult SES, and cognitive decline by comparing different life course models within the same analytical sample. By identifying points in childhood and adulthood at which interventions might be most effective in regard to modifying the accumulation of risk factors, this may help to prevent cognitive decline.

## Data Availability Statement

The data analyzed in this study is subject to the following licenses/restrictions: Due to ethical restrictions, data are available upon request from the Lothian Birth Cohort 1936 Study. Requests to access these datasets should be directed to Simon Cox, simon.cox@ed.ac.uk.

## Ethics Statement

The studies involving human participants were reviewed and approved by Ethics permission for the Lothian Birth Cohort 1936 was obtained from the Multi-Centre Research Ethics Committee for Scotland (wave 1:MREC/01/0/56), the Lothian Research Ethics Committee (wave 1: LREC/2003/2/29), and the Scotland A Research Ethics Committee (waves 2 and 3: 07/MRE00/58). The patients/participants provided their written informed consent to participate in this study.

## Author Contributions

SRM contributed to the conception and design of the study, to conducting the literature review, performing the analysis and the interpretation of data, and wrote the paper. AH contributed to the design of the study, conducting the literature review and content. VT contributed to the design of the study, reviewed the original content and critically revised it for important intellectual content. OP contributed to performing the analysis and the interpretation of data. CH, a senior author, reviewed the paper and revised it critically for important intellectual content. SD, a senior author, reviewed the paper and revised it critically for important intellectual content, and contributed to the acquisition of data. All authors contributed to the article and approved the submitted version.

### Conflict of Interest

The authors declare that the research was conducted in the absence of any commercial or financial relationships that could be construed as a potential conflict of interest.

## Abbreviations

SES: Socioeconomic status
LBC1936: The Lothian Birth Cohort 1936
